# Interval oscillation criteria for second-order forced impulsive delay differential equations with damping term

**DOI:** 10.1186/s40064-016-2117-5

**Published:** 2016-05-04

**Authors:** Ethiraju Thandapani, Manju Kannan, Sandra Pinelas

**Affiliations:** Ramanujan Institute for Advanced Study in Mathematics, University of Madras, Chennai, 600 005 India; Departamento De Ciencias Exactas E Naturais, Academia Militar, Av. Conde Castro Guimaraes, 2720-113 Amadora, Portugal

**Keywords:** Oscillation, Second-order, Impulse, Damping term, Differential equation, 34A37, 34C10

## Abstract

In this paper, we present some sufficient conditions for the oscillation of all solutions of a second order forced impulsive delay differential equation with damping term. Three factors-impulse, delay and damping that affect the interval qualitative properties of solutions of equations are taken into account together. The results obtained in this paper extend and generalize some of the the known results for forced impulsive differential equations. An example is provided to illustrate the main result.

## Background

In this paper, we consider the second-order impulsive differential equation with mixed nonlinearities of the form

1 $$\begin{aligned} \left\{ \begin{array}{ll} (r(t)(x'(t))^{\gamma })'+p(t)(x'(t))^{\gamma }+q(t)x^{\gamma }(t-\delta )\\ \quad\quad\quad\quad\quad +\sum \nolimits _{i=1}^{n}q_i(t)|x(t-\delta )|^{\alpha _i-1} x(t-\delta )=e(t),\quad \, t\ne \tau _k;\\ x(\tau _k^+)=a_kx(\tau _k), \quad x^{'}(\tau _k^+)=b_kx'(\tau _k) \end{array} \right. \end{aligned}$$

where $t\ge t_0,\, k\in {\mathbb {N}}, \{\tau _k\}$ is the impulse moments sequence with

$$\begin{aligned} 0\le t_0=\tau _0<\tau _1\ldots ,\quad  \lim \limits _{t\rightarrow \infty }\tau _k=\infty , \end{aligned}$$

and

$$\begin{aligned} x(\tau _k)&=  x(\tau _k^-)=\lim \limits _{t\rightarrow \tau _k^{-}}x(t),\,\,x(\tau _k^+)=\lim \limits _{t\rightarrow \tau _k^{+}}x(t)\\ x'(\tau _k)&= x'(\tau _k^-)=\lim \limits _{h\rightarrow 0^-}\frac{x(\tau _k+h)-x(\tau _k)}{h},\\ x'(\tau _k^+)& = \lim \limits _{h\rightarrow 0^+}\frac{x(\tau _k+h)-x(\tau _k^+)}{h}. \end{aligned}$$

Let $J\subset {\mathbb {R}}$ be an interval and define $PLC(J, {\mathbb {R}})=\{x:J\rightarrow {\mathbb {R}}:x(t)$ is continuous on each interval $(\tau _k,\tau _{k+1}),\, x(\tau _k^{\pm })$ exist, and $x(\tau _k)=x(\tau _k^{-})$ for all $k\in {\mathbb {N}}\}.$

For given $t_0$ and $\phi \in PLC([t_0-\delta ,t_0], {\mathbb {R}}),$ we say $x \in PLC([t_0-\delta ,\infty ], {\mathbb {R}})$ is a solution of Eq. (1) with initial value $\phi$ if *x*(*t*) satisfies (1) for $t\ge t_0$ and $x(t)=\phi (t)$ for all $t\in [t_0-\delta ,t_0] .$ A non-trivial solution is called oscillatory if it has infinitely many zeros;otherwise it is called non-oscillatory.

In recent years the theory of impulsive differential equations emerge as an important area of research, since such equations have applications in the control theory, physics, biology, population dynamics, economics, etc. For further applications and questions concerning existence and uniqueness of solutions of impulsive differential equation, see Bainov and Simenov (1993), Lakshmikantham et al. (1989). In the last decades, interval oscillation of impulsive differential equations was arousing the interest of many researchers, see Li and Cheung (2013), Liu and Xu (2007, 2009), Muthulakshmi and Thandapani (2011) and Özbekler and Zafer (2009, 2011) considered the following equations

2 $$\begin{aligned} \left\{ \begin{array}{ll} (r(t)(\Phi _{\alpha }(x'))'+p(t)\Phi _{\alpha }(x')+q(t)\Phi _{\beta }(x)=e(t),&{}\quad t\ne \tau _k;\\ \Delta (r(t)\Phi _{\alpha }(x'))+q_i\Phi _{\beta }(x)=e_i, &{}\quad t= \tau _k,\, k\in {\mathbb {N}}. \end{array} \right. \end{aligned}$$

As far as we know, it is the first article focusing on the interval oscillation for the impulsive differential equation with damping term. Their results well improved and extended the earlier one for the equations without impulse or damping. Recently Guo et al. (2014) considered a class of second order nonlinear impulsive delay differential equations with damping term and established some interval oscillation criteria for that equation.

However, for the impulsive equations, almost all of interval oscillation results in the existing literature were established only for the case of “without delay”, in other words, for the case of “with delay” the study on the interval oscillation is very scarce. To the best of our knowledge, Huang and Feng (2010) gave the first research in this direction recently. They considered second order delay differential equations with impulses

3 $$\begin{aligned} \left\{ \begin{array}{ll} x''(t)+p(t)f(x(t-\tau ))=e(t),\quad t\ge t_0,\quad  t\ne t_k;\\ \\ x(t_k^+)=a_kx(t_k),\quad  x^{'}(t_k^+)=b_kx'(t_k),\quad  \, k=1,2,\ldots \end{array} \right. \end{aligned}$$

and established some interval oscillation criteria which developed some known results for the equations without delay or impulses (Liu and Xu 2007; El Sayed 1993). It is natural to ask if it is possible to research the interval oscillation of the impulsive delay equations with damping term. In this paper, motivated mainly by Huang and Feng (2010) and Özbekler and Zafer (2009), we study the interval oscillation of second order nonlinear impulsive delay differential equations with damping term (1). We establish some interval oscillation criteria which generalize or improve some known results of Guo et al. (2012a, b, 2014), Liu and Xu (2007, 2009), Muthulakshmi and Thandapani (2011), Pandian and Purushothaman (2012), Özbekler and Zafer (2009, 2011) and Li and Cheung (2013). Finally we give an example to illustrate our main result.

## Main results

Throughout this paper, we assume that the following conditions hold:

1. $r(t)\in C^{1}([t_0,\infty ),(0,\infty ))$ and $p(t),\,q(t),\, q_i(t),\, e(t)\in PLC([t_0,\infty ), {\mathbb {R}}),\,i=1,2 \ldots , n,$ with $r'(t)+p(t)\ge 0$ for all $t\ge t_o;$
2. $$\delta \ge 0,\,\tau _{k+1}-\tau _k>\delta ,\,k\in {\mathbb {N}},\ \alpha _1>\cdots>\alpha _m>\gamma>\alpha _{m+1}>\cdots>\alpha _n>0$$ are constants;
3. $$ a_k,b_k$$ are real constants satisfying $$b_k\ge a_k>0,\, k=1,2, \ldots .$$

We begin with the following notations: $I(s)=\max \{i:t_0<\tau _i<s\},\, r_j=\max \{r(t):t\in [c_j,d_j]\},\,j=1,2$ and

$$\begin{aligned} E_{c_j,d_j}=\{u\in C^{1}([c_j,d_j],{\mathbb {R}}):\ u(t)\not \equiv 0,\,u(c_j)=u(d_j)=0\}. \end{aligned}$$

For two constants $c,d \notin \{\tau _k\}$ with $c<d$ and a function $\varphi \in C([c,d],{\mathbb {R}}),$ we define an operator $\Omega :C([c,d],{\mathbb {R}})\rightarrow {\mathbb {R}}$ by

$$\Omega _c^d[\varphi ]=\left\{ \begin{array}{ll} 0 &\quad  \text{ for }\,\,\ I(c)=I(d), \\ \varphi (\tau _{I(c)+1})\theta (c)+\sum \nolimits _{i=I(c)+2}^{I(d)}\varphi (\tau _i)\varepsilon (\tau _i) &\quad \text{ for } \,\,\ I(c)<I(d), \\ \end{array}\right.$$

where

$$\theta (c)=\frac{(a_{I(c)+1})^{\gamma }-(b_{I(c)+1})^{\gamma }}{(a_{I(c)+1})^\gamma (\tau _{I(c)+1}-c)^\gamma }\quad \text{ and } \quad\varepsilon (\tau _i) =\frac{a_i^\gamma -b_i^\gamma }{a_i^\gamma (\tau _i-\tau _{i-1})^\gamma }.$$

To prove our main results, we need the following lemmas.

### **Lemma 1**

*Let*$(\alpha _1,\alpha _2,\ldots , \alpha _n)$*be ann*-*tuple satisfying*$\alpha _1> \alpha _2>\cdots> \alpha _m>\gamma> \alpha _{m+1}>\cdots>\alpha _n>0.$*Then there exists ann*-*tuple*$(\eta _1, \eta _2,\ldots ,\eta _n)$*satisfying*

4 $$\begin{aligned} \sum \limits _{i=1}^{n}\alpha _i\eta _i=\gamma \end{aligned}$$

*and also either*

5 $$\begin{aligned} \sum \limits _{i=1}^{n}\eta _i<1,\quad  0<\eta _i<1 \end{aligned}$$

or

6 $$\begin{aligned} \sum \limits _{i=1}^{n}\eta _i=1,\quad 0<\eta _i<1. \end{aligned}$$

The proof of Lemma 1 can be found in Hassan et al. (2011) and Özbekler and Zafer (2011) which is the extension of (Lemma 1, Sun and Wong 2007).

### *Remark 1*

For given constants $\alpha _1> \alpha _2>\ldots \alpha _m>\gamma> \alpha _{m+1}>\cdots>\alpha _n>0,$ Lemma 1 ensures the existence of *n*-tuple $(\eta _1, \eta _2,\ldots ,\eta _n)$ such that either (4) and (5) or (4) and (6) hold. Particularly when $n=2,$ and $\alpha _1>\gamma>\alpha _2>0$ in the first case we have

$$\eta _1=\frac{\gamma -\alpha _2(1-\eta _0)}{\alpha _1-\alpha _2},\quad \eta _2=\frac{\alpha _1(1-\eta _0)-\gamma }{\alpha _1-\alpha _2} $$

where $\eta _0$ be any positive number satisfying $0<\eta _0<\frac{\alpha _1-\gamma }{\alpha _1}.$ This will ensure that $0<\eta _1,\eta _2<1$ and conditions (4) and (5) are satisfied. In the second case, we can solve (4) and (6) and obtain

$$ \eta _1=\frac{\gamma -\alpha _2}{\alpha _1-\alpha _2},\quad \eta _2=\frac{\alpha _1-\gamma }{\alpha _1-\alpha _2}.$$

The Lemma below can be found in Hardy et al. (1934).

### **Lemma 2**

*LetXandYbe non-negative real numbers. Then*

$$\begin{aligned} \lambda XY^{\lambda -1}-X^\lambda \le (\lambda -1)Y^{\lambda },\quad \,\lambda >1 \end{aligned}$$

*where equality holds if and only if*$X=Y.$

*Let*$\gamma>0,\,A\ge 0,\, B>0$*and*$y>0.$*Put*$\lambda =1+\frac{1}{\gamma },\, X=B^{\frac{\gamma }{\gamma +1}}y,\, Y=\left( \frac{\gamma }{\gamma +1}\right) ^\gamma A^\gamma B^{\frac{-\gamma ^2}{\gamma +1}}$*in Lemma* 2, *we have*

7 $$\begin{aligned} A-B\le \left( \frac{A}{\gamma +1}\right) ^{\gamma +1}\left( \frac{\gamma }{B}\right) ^{\gamma }. \end{aligned}$$

### **Theorem 1**

*Suppose that for any*$T>0,$*there exist*$c_j,d_j\notin \{\tau _k\},\, j=1,2$*such that*$c_1<d_1\le d_1+\delta \le c_2<d_2$*and*$q(t),\, q_i(t)\ge 0,\, t\in [c_1-\delta ,d_1]\ \cup \ [c_2-\delta , d_2], i=1,2, \ldots, n$*and*

8 $$\begin{aligned} e(t)=\left\{ \begin{array}{ll} \le 0 &\quad {\textit{if}}\ \ t\in [c_1-\delta , d_1], \\ \ge 0 &\quad {\textit{if}} \ \ t\in [c_2-\delta , d_2], \\ \end{array}\right. \end{aligned}$$

*and*$u_j\in E_{c_j,d_j}$*such that*

9 $$\begin{aligned} &\int _{c_j}^{d_j}\Bigg [\frac{r(t)}{(\gamma +1)^{\gamma +1}}\Big |(\gamma +1)u'(t)-\frac{p(t)u(t)}{r(t)}\Big |^{\gamma +1}\Bigg ]dt- \int ^{\tau _{_{I(c_j)+1}}}_{c_j}Q(t)Q^{j}_{I(c_j)}(t)|u(t)|^{\gamma +1}dt\nonumber \\ & \quad\quad -\sum \limits _{k=I(c_j)+2}^{I(d_j)}\int _{\tau _{_{k-1}}}^{\tau _{_{k}}}Q(t)Q_k^j(t) |u(t)|^{\gamma +1}dt -\int _{\tau _{_{I(d_j)}}}^{d_j}Q(t)Q_{I(d_j)}^j(t)|u(t)|^{\gamma +1}dt\nonumber \\ & \quad < r_j \Omega _{c_j}^{d_j}[|u(t)|^{\gamma +1}],\quad \, j=1,2 \end{aligned}$$

*where*

$$\begin{aligned} Q(t)=q(t)+\eta _{0}^{-\eta _0}\prod \limits _{i=1}^{n}(\eta _{i}^{-1}q_i(t))^{\eta _{i}}|e(t)|^{\eta _0},\quad \eta _0=1-\sum \limits _{i=1}^{n}\eta _i \end{aligned}$$

*where*$\eta _i>0$*are chosen according to given*$\alpha _1, \alpha _2, \ldots \alpha _n$*as in Lemma* 1 *satisfying* (4) *and* (5), *and*

$$\begin{aligned} Q_{k}^{j}(t)=\left\{ \begin{array}{cc} \frac{(t-\tau _k)^{\gamma }}{(a_k\delta +b_k(\tau -\tau _k))^\gamma }, &{}\ t\in (\tau _k,\tau _k+\delta ), \\ \frac{(\tau -\tau _k-\delta )^\gamma }{(\tau -\tau _k)^\gamma } , &{} \ t\in [\tau _k+\delta ,\tau _{k+1}], \\ \end{array}\right. k=I(c_j),I(c_j)+1,\ldots ,I(d_j), \end{aligned}$$

*then every solution of Eq.* (1) *is oscillatory.*

### *Proof*

Let *x*(*t*) be a non-oscillatory solution of Eq. (1). Without loss of generality, we may assume that $$x(t)>0 $$ and $$x(t-\delta )>0$$ for all $$t\ge t_0>0.$$ Define

10 $$\begin{aligned} \omega (t)=\frac{r(t)(x'(t))^{\gamma }}{x^{\gamma }(t)},\quad t\in [c_1-\delta ,d_1]. \end{aligned}$$

Then for all $t\ne \tau _k,\,t\ge t_0,$ we have

11 $$\begin{aligned} \omega '(t)=-q(t)\frac{x^{\gamma }(t-\delta )}{x^{\gamma }(t)}-\sum \limits _{i=1}^{n}q_i(t)|x(t-\delta )|^{\alpha _i-\gamma }\frac{x^{\gamma }(t-\delta )}{x^{\gamma }(t)} +\frac{e(t)}{x^{\gamma }(t)}-\frac{p(t)\omega (t)}{r(t)}-\frac{\gamma |\omega (t)|^{\frac{\gamma +1}{\gamma }}}{(r(t))^{\frac{1}{\gamma }}}. \end{aligned}$$

By taking $$\eta _0:=1-\sum \nolimits _{i=1}^{n}\eta _i,$$

$$\begin{aligned} \zeta _0& =  \eta _0^{-1}\left| \frac{e(t)x^{\gamma }(t-\delta )}{x^{\gamma }(t)}\right| x^{-\gamma }(t-\delta )\\ \zeta _i &= \eta _i^{-1}q_i(t)\frac{x^{\gamma }(t-\delta )}{x^{\gamma }(t)}x^{\alpha _i-\gamma }(t-\delta ),\quad i=1,2, \ldots , n \end{aligned}$$

and using the the arithmetic–geometric mean inequality,

$$\begin{aligned} \sum \limits _{i=0}^{n}\eta _i\zeta _i\ge \prod \limits _{i=0}^{n}\zeta _i^{\eta _i},\,\, \zeta _i\ge 0 \end{aligned}$$

we have

12 $$\begin{aligned} \sum \limits _{i=1}^{n}q_i(t)\frac{x^{\alpha _i-\gamma }(t-\delta )}{x^{\gamma }(t)}x^{\gamma }(t-\delta )+\frac{|e(t)|}{x^{\gamma }(t)}\ge& \,\eta _0^{-\eta _0}|e(t)|^{\eta _0} \prod \limits _{i=0}^{n}\eta _{i}^{-\eta _i}q_i^{\eta _i}(t)\frac{x^{\eta _i(\alpha _i-\gamma )}(t-\delta )}{x^{\eta _i\gamma }(t)}x^{\eta _i\gamma }(t-\delta )\nonumber \\ &\times \frac{x^{\eta _{_0}\gamma }(t-\delta )}{x^{\eta _{_0}\gamma }(t)}x^{-\eta _{_0}\gamma }(t-\delta ). \end{aligned}$$

Since

$$\begin{aligned} \prod \limits _{i=0}^{n}\frac{x^{\eta _{i}\gamma }(t-\delta )}{x^{\eta _i\gamma }(t)}=\frac{x^{(\eta _{_0}+\eta _{_1}+\cdots +\eta _{_n})\gamma }(t-\delta )}{x^{(\eta _{_0}+\eta _{_1}+\cdots +\eta _{_n})\gamma }(t)}= \frac{x^{\gamma }(t-\delta )}{x^{\gamma }(t)} \end{aligned}$$

and

$$\begin{aligned} \prod \limits _{i=1}^{n}x^{(\alpha _i-\gamma )\eta _{_i}}(t-\delta )x^{-\eta _{_0}\gamma }(t-\delta )=1, \end{aligned}$$

from (12), (11) becomes

13 $$\begin{aligned} \omega '(t)\le & {} -q(t)\frac{x^{\gamma }(t-\delta )}{x^{\gamma }(t)}-\eta _{_0}^{-\eta _{_0}}\prod \limits _{i=1}^{n}\eta _{_i}^{-\eta _{_i}} q_i^{\eta _{_i}}(t)|e(t)|^{\eta _{_0}}-\frac{p(t)\omega (t)}{r(t)} -\frac{\gamma |\omega (t)|^{\frac{\gamma +1}{\gamma }}}{(r(t))^{\frac{1}{\gamma }}} \nonumber \\= & {} -Q(t)\frac{x^{\gamma }(t-\delta )}{x^{\gamma }(t)}-\frac{p(t)\omega (t)}{r(t)} -\frac{\gamma |\omega (t)|^{\frac{\gamma +1}{\gamma }}}{(r(t))^{\frac{1}{\gamma }}},\quad t\ne \tau _k. \end{aligned}$$

For $t=\tau _k,\, k=1,2,\ldots ,$ we have

14 $$\begin{aligned} \omega (\tau _k^+)=\frac{b_k^{\gamma }}{a_k^{\gamma }}\omega (\tau _k). \end{aligned}$$

Multiply both sides of (13) by $|u(t)|^{\gamma +1}$ where $u(t)\in E_{c_{_{1}}, d_{_{1}}}$ and integrating from $c_1$ to $d_1,$ then using integration by parts on the left side, we have

15 $$\begin{aligned}&\sum \limits _{k=I(c_1)+1}^{I(d_1)}|u(\tau _k)|^{\gamma +1}[\omega (\tau _k)-\omega (\tau _k^+)] \nonumber \\ & \quad \le \int _{c_1}^{d_1}(\gamma +1)u^{\gamma }(t)u'(t)\omega (t)dt-\int _{c_1}^{d_1} Q(t)|u(t)|^{\gamma +1}\frac{x^{\gamma }(t-\delta )}{x^{\gamma }(t)}dt\nonumber \\ & \quad\quad-\int _{c_1}^{d_1}\frac{p(t)\omega (t)}{r(t)}|u(t)|^{\gamma +1}dt -\int _{c_1}^{d_1}\frac{\gamma |\omega (t)|^{\frac{\gamma +1}{\gamma }}}{(r(t))^{\frac{1}{\gamma }}}|u(t)|^{\gamma +1}dt\nonumber \\ &\quad \le -\int _{c_1}^{\tau _{_{I(c_1)+1}}} Q(t)|u(t)|^{\gamma +1}\frac{x^{\gamma }(t-\delta )}{x^{\gamma }(t)}dt-\sum \limits _{k=I(c_1)+1}^{I(d_1-1)} \int _{\tau _k}^{\tau _{k+1}} Q(t)|u(t)|^{\gamma +1}\frac{x^{\gamma }(t-\delta )}{x^{\gamma }(t)}dt \nonumber \\ &\quad \quad-\int _{\tau _{_{I(d_1)}}}^{d_1}Q(t)|u(t)|^{\gamma +1}\frac{x^{\gamma }(t-\delta )}{x^{\gamma }(t)}dt+\int _{c_1}^{d_1}\Bigg [\left( \Bigg |(\gamma +1)u'(t) -\frac{p(t)u(t)}{r(t)}\Bigg |\right) |\omega (t)||u(t)|^{\gamma } \nonumber \\ &\quad\quad -\frac{\gamma |\omega (t)|^{\frac{\gamma +1}{\gamma }}}{(r(t))^{\frac{1}{\gamma }}}|u(t)|^{\gamma +1}\Bigg ]dt. \end{aligned}$$

Using (7) with

$$\begin{aligned} A=\left( \Bigg |(\gamma +1)u'(t) -\frac{p(t)u(t)}{r(t)}\Bigg |\right) ,\quad B=\frac{\gamma }{(r(t))^{\frac{1}{\gamma }}},\,\ \text{ and }\ y=|\omega (t)||u(t)|^{\gamma } \end{aligned}$$

we have

16 $$\begin{aligned} & \left( (\gamma +1)|u'(t)| -\frac{p(t)|u(t)|}{r(t)}\right)|\omega (t)||u(t)|^{\gamma } -\frac{\gamma |\omega (t)|^{\frac{\gamma +1}{\gamma }}}{(r(t))^{\frac{1}{\gamma }}}|u(t)|^{\gamma +1}\nonumber \\ & \quad\quad \le \frac{r(t)}{(\gamma +1)^{\gamma +1}}\Big ((\gamma +1)|u'(t)|-\frac{p(t)|u(t)|}{r(t)}\Big )^{\gamma +1}. \end{aligned}$$

Now for $t\in [c_1,d_1]\setminus {\tau _k},\, k\in {\mathbb {N}}$ from (1) it is clear that

$$\begin{aligned} (r(t)(x'(t))^{\gamma })'+p(t)(x'(t))^{\gamma }=e(t)-q(t)x^{\gamma }(t-\delta )-\sum \limits _{i=1}^{n}q_i(t)|x(t-\delta )|^{\alpha _i-1} x(t-\delta )\le 0. \end{aligned}$$

That is

$$\begin{aligned} ((x'(t))^{\gamma })'+\Big (\frac{r'(t)+p(t)}{r(t)}\Big )(x'(t))^{\gamma }\le 0 \end{aligned}$$

which implies that

$$\begin{aligned} (x'(t))^{\gamma }\exp \int _{c_1}^{t}\frac{r'(s)+p(s)}{r(s)}ds \end{aligned}$$

is non-increasing on $[c_1,d_1]\setminus {\tau _k}.$

Because there are different integration intervals in (15), we will estimate $x(t-\delta )/x(t)$ in each interval of *t* as follows. We first consider the situation where $I(c_1)\le I(d_1).$ In this case, all the impulsive moments in $[c_1,d_1]$ are $\tau _{I(c_1)+1},\, \tau _{I(c_2)+1},\, \ldots \tau _{I(d_1)}.$

- **Case 1** For $t\in (\tau _k, \tau _{k+1}]\subset [c_1, d_1]$ we have the following two sub cases:
  - If $\tau _k+\delta \le t\le \tau _{k+1},$ then $(t-\delta , t)\subset (\tau _k, \tau _{k+1}].$ Thus there is no impulse moment in $(t-\delta , t).$ For any $s \in (t-\delta , t),$ we have $x(s)>x(s)-x(\tau _k^+)=x'(\xi )(s-\tau _k),\quad \xi \in (\tau _k,s).$ Then
    17 $$\begin{aligned} (x(s))^\gamma \ge (x'(\xi ))^\gamma (s-\tau _k)^\gamma . \end{aligned}$$
    Since $(x'(s))^{\gamma }\exp \int _{c_1}^{s}\frac{r'(v)+p(v)}{r(v)}dv$ is non-increasing in $[c_1,t],$ we have
    18 $$\begin{aligned} (x'(\xi ))^{\gamma }\exp \int _{c_1}^{\xi }\frac{r'(v)+p(v)}{r(v)}dv\ge (x'(s))^{\gamma }\exp \int _{c_1}^{s}\frac{r'(v)+p(v)}{r(v)}dv. \end{aligned}$$
    From (17) and (18) we have
    19 $$\begin{aligned} (x(s))^\gamma\ge & \, \frac{(x'(s))^{\gamma }\exp \int _{c_1}^{s}\frac{r'(v)+p(v)}{r(v)}dv}{\exp \int _{c_1}^{\xi }\frac{r'(v)+p(v)}{r(v)}dv}(s-\tau _k)^{\gamma } \nonumber \\\ge & \, (x'(s))^\gamma (s-\tau _k)^{\gamma }. \end{aligned}$$
    Therefore $\frac{x'(s)}{x(s)}<\frac{1}{s-\tau _k}.$ Integrating both sides of the above inequality from $t-\delta$ to *t*,  we obtain
    $$\begin{aligned} \frac{x(t-\delta )}{x(t)}>\frac{t-\tau _k-\delta }{t-\tau _k}>0. \end{aligned}$$
  - If $\tau _k<t<\tau _k+\delta ,$ then $\tau _k-\delta<t-\delta<\tau _k<t <\tau _{k}+\delta .$ There is an impulsive moment $\tau _k$ in $(t-\delta ,t).$ Similar to (a), we have $\frac{x'(s)}{x(s)}<\frac{1}{s-\tau _k+\delta }$ for any $s\in (\tau _k-\delta , \tau _k].$ Upon integrating from $t-\delta$ to $\tau _k,$ we obtain
    20 $$\begin{aligned} \frac{x(t-\delta )}{x(\tau _k)} >\frac{t-\tau _k}{\delta }\ge 0. \end{aligned}$$
    For any $t\in (\tau _k,\tau _k+\delta ),$ we have
    $$\begin{aligned} x(t)-x(\tau _k^+)<x'(t_k^+)(t-\tau _k),\,\, \xi \in (\tau _k,t). \end{aligned}$$
    Using the impulsive conditions in Eq. (1) we get
    $$\begin{aligned} x(t)-a_k x(\tau _k) & <   b_k x'(\tau _k)(t-\tau _k)\\ \frac{x(t)}{x(\tau _k)} & \le  \frac{b_k x'(\tau _k)}{x(\tau _k)}(t-\tau _k)+a_k. \end{aligned}$$
    Using $\frac{x'(\tau _k)}{x(\tau _k)}<\frac{1}{\delta },$ we obtain
    $$\begin{aligned} \frac{x(t)}{x(\tau _k)}<a_k+\frac{b_k}{\delta }(t-\tau _k). \end{aligned}$$
    That is
    21 $$\begin{aligned} \frac{x(\tau _k)}{x(t)}>\frac{\delta }{a_k\delta +b_k(t-\tau _k)}. \end{aligned}$$
    From (20) and (21), we have
    $$\begin{aligned} \frac{x(t-\delta )}{x(t)}>\frac{t-\tau _k}{a_k\delta +b_k(t-\tau _k)}\ge 0. \end{aligned}$$

- **Case 2** For $t\in [c_1,\tau _{_{I(c_1)+1}})$ we have the following three sub-cases:
  - If $c_1<t<\tau _{_{I(c_1)}}+\delta$ and $\tau _{_{I(c_1)}}>c_1-\delta ,$ then $t-\delta \in [c_1-\delta , \tau _{_{I(c_1)}})$ and there is an impulsive moment $\tau _{_{I(c_1)}}$ in $(t-\delta ,t).$ Similar to Case 1(b), we have
    $$ \frac{x(t-\delta )}{x(t)}> \frac{t-\tau _{_{I(c_1)}}}{a_{_{I(c_1)}}\delta +b_{_{I(c_1)}}(t-\tau _{_{I(c_1)}})}\ge 0.$$
  - If $\tau _{_{I(c_1)}} + \tau < t <\tau _{_{I(c_1)+1}}$ and $\tau_{_{I(c_1)}}>c_1-\delta,$ then there are no impulsive moments in $(t-\delta ,t).$ Making a similar analysis of Case 1(a), we obtain $\frac{x(t-\delta )}{x(t)}> \frac{t-\delta -\tau _{_{I(c_1)}}}{t-\tau _{_{I(c_1)}}}\ge 0.$
  - If $\tau _{_{I(c_1)}}>c_1-\delta ,$ then there are no impulsive moments in $(t-\delta ,t).$ So
    $$\begin{aligned} \frac{x(t-\delta )}{x(t)}> \frac{t-\delta -\tau _{_{I(c_1)}}}{t-\tau _{_{I(c_1)}}}\ge 0. \end{aligned}$$

- **Case 3** For $t\in (\tau _{_{I(d_1)}}, d_1],$ there are three sub-cases:
  - If $\tau _{_{I(d_1)}}+\delta <d_1,\,\ t\in [\tau _{_{I(d_1)}},\tau _{_{I(d_1)}}+\delta ),$ then there is an impulsive moment $\tau _{_{I(d_1)}}.$ Similar to Case 2(a), we have
    $$\begin{aligned} \frac{x(t-\delta )}{x(t)}> \frac{t-\tau _{_{I(d_1)}}}{a_{_{I(d_1)}}\delta +b_{_{I(d_1)}}(t-\tau _{_{I(d_1)}})}\ge 0. \end{aligned}$$
  - If $\tau _{_{I(d_1)}}+\delta<t<d_1$ then there are no impulsive moments in $(t-\delta ,t).$ Making a similar analysis of Case 2(b), we obtain
    $$\begin{aligned} \frac{x(t-\delta )}{x(t)}> \frac{t-\delta -\tau _{_{I(d_1)}}}{t-\tau _{_{I(d_1)}}}\ge 0. \end{aligned}$$
  - If $\tau _{_{I(d_1)}}+\delta \ge d_1,$ then there is an impulsive moment $\tau _{_{I(d_1)}}$ in $(t-\delta ,t).$

Similar to Case 3(a), we obtain

$$\begin{aligned} \frac{x(t-\delta )}{x(t)}> \frac{t-\tau _{_{I(d_1)}}}{a_{_{I(d_1)}}\delta +b_{_{I(d_1)}}(t-\tau _{_{I(d_1)}})}\ge 0. \end{aligned}$$

Combining all these cases, we have

22 $$\begin{aligned} \frac{x^\gamma (t-\delta )}{x^\gamma (t)}>\left\{ \begin{array}{ll} Q_{_{I(c_1)}}^{1}(t)&{} \ \text{ for }\quad \ t\in [c_1,\tau _{_{I(c_1)+1}}],\\ Q_{k}^{1}(t)&{} \ \text{ for }\quad t\in (\tau _k,\tau _{k+1}],\, k=I(c_1)+1,\ldots ,I(d_1)-1,\\ Q_{_{I(d_1)}}^{1}(t) &{}\ \text{ for }\quad t\in (\tau _{_{I(d_1)+1}},d_1]\\ \end{array}\right. \end{aligned}$$

Using (16) and (22) in (15) we get

23 $$\begin{aligned}&\sum \limits _{k=I(c_1)+1}^{I(d_1)}|u(\tau _k)|^{\gamma +1}[\omega (\tau _k)-\omega (\tau _k^+)] \nonumber \\ &\quad \le \int _{c_1}^{d_1} \frac{r(t)}{(\gamma +1)^{\gamma +1}}\Big ((\gamma +1)|u'(t)|-\frac{p(t)|u(t)|}{r(t)}\Big )^{\gamma +1}dt- \int _{c_1}^{\tau _{I(c_1)+1}} Q(t)|u(t)|^{\gamma +1}Q_{I(c_1)}^1(t)dt\nonumber \\ &\quad \quad -\sum \limits _{k=I(c_1)+1}^{I(d_1-1)} \int _{\tau _k}^{\tau _{k+1}} Q(t)|u(t)|^{\gamma +1}Q_{k}^{1}(t)dt -\int _{\tau _{I(d_1)}}^{d_1}Q(t)|u(t)|^{\gamma +1}Q_{_{I(d_1)}}^{1}(t)dt. \end{aligned}$$

For any $t \in (c_1,\tau _{_{I(c_1)+1}}],$ we have $x(t)-x(c_1)=x'(\xi )(t-c_1),\, \xi \in (c_1,t).$ Since $x(c_1)>0,$ we have $x(t)>x'(\xi )(t-c_1).$ Then

24 $$(x(t))^\gamma >(x'(\xi ))^\gamma (t-c_1)^\gamma. $$

Using the monotonicity of $(x'(t))^{\gamma }\exp \left( \int _{c_1}^{t}\frac{r'(s)+p(s)}{r(s)}ds\right) ,$ and (24) we have

$$\begin{aligned} (x(t))^\gamma &\ge \frac{(x'(t))^{\gamma }\exp \left( \int _{c_1}^{t}\frac{r'(s)+p(s)}{r(s)}ds\right) }{\exp \left( \int _{c_1}^{\xi }\frac{r'(s)+p(s)}{r(s)}ds\right) }(t-c_1)^\gamma \\ &\ge (x'(t))^{\gamma }(t-c_1)^\gamma \end{aligned}$$

for some $\xi \in (c_1,t).$ It follows

$$\begin{aligned} \frac{(x'(t))^{\gamma }}{(x(t))^\gamma }\le \frac{1}{(t-c_1)^\gamma }. \end{aligned}$$

Letting $t\rightarrow \tau _{_{I(c_1)+1}},$ from (9), we have

25 $$\begin{aligned} \omega (\tau _{_{I(c_1)+1}})\le \frac{r_1}{(\tau _{_{I(c_1)+1}}-c_1)^\gamma }. \end{aligned}$$

Making a similar analysis on $(\tau _{k-1}, \tau _k],\, k=I(c_1)+2,\ldots ,I(d_1),$ we can prove that

26 $$\begin{aligned} \omega (\tau _k)\le \frac{r_1}{(\tau _{k}-\tau _{k-1})^\gamma }. \end{aligned}$$

From (24), (25) and (A3), we obtain

27 $$\begin{aligned}&\sum \limits _{k=I(c_1)+1}^{I(d_1)}\frac{a_k^\gamma -b_k^\gamma }{a_k^\gamma }|u(\tau _k)|^{\gamma +1}\omega (\tau _k)\nonumber \\ & \quad \ge  \frac{a_{I(c_1)+1}^\gamma -b_{I(c_1)+1}^\gamma }{a_{I(c_1)+1}^\gamma (\tau _{_I(c_1)+1}-c_1)^\gamma }|u(\tau _{_{I(c_1)+1}})|^{\gamma +1}r_1+ \sum \limits _{k=I(c_1)+2}^{I(d_1)}\frac{a_k^\gamma -b_k^\gamma }{a_k^\gamma (\tau _k-\tau _{k-1})^{\gamma }}|u(\tau _k)|^{\gamma +1}r_1\nonumber \\ &\quad =   r_1 \Omega _{c_1}^{d_1}[|u(t)|^{\gamma +1}]. \end{aligned}$$

Since

$$\begin{aligned} \sum \limits _{k=I(c_1)+2}^{I(d_1)}|u(\tau _k)|^{\gamma +1}[\omega (\tau _k)-\omega (\tau _k^+)] =\sum \limits _{k=I(c_1)+1}^{I(d_1)}\frac{a_k^\gamma -b_k^\gamma }{a_k^\gamma }|u(\tau _k)|^{\gamma +1}\omega (\tau _k), \end{aligned}$$

from (23) we have

$$\begin{aligned}&\int _{c_1}^{d_1} \frac{r(t)}{(\gamma +1)^{\gamma +1}}\Big ((\gamma +1)|u'(t)|-\frac{p(t)|u(t)|}{r(t)}\Big )^{\gamma +1}dt- \int _{c_1}^{\tau _{I(c_1)+1}} Q(t)|u(t)|^{\gamma +1}Q_{I(c_1)}^1(t)dt\nonumber \\ &\quad -\sum \limits _{k=I(c_1)+2}^{I(d_1)-1} \int _{\tau _{k-1}}^{\tau _{k}} Q(t)|u(t)|^{\gamma +1}Q_{k}^{1}(t)dt -\int _{\tau _{I(d_1)}}^{d_1}Q(t)|u(t)|^{\gamma +1}Q_{_{I(d_1)}}^{1}(t)dt>r_1 \Omega _{c_1}^{d_1}[|u(t)|^{\gamma +1}] \end{aligned}$$

which contradicts (9).

If $I(c_1)=I(d_1),$ then $\Omega _{c_1}^{d_1}[|u(t)|^{\gamma +1}]=0$ and there is no impulsive moments in $[c_1,d_1].$ Similar to the proof of (22), we obtain

$$\begin{aligned} \int _{c_1}^{d_1} \frac{r(t)}{(\gamma +1)^{\gamma +1}}\Big ((\gamma +1)|u'(t)|-\frac{p(t)|u(t)|}{r(t)}\Big )^{\gamma +1}dt- \int _{c_1}^{\tau _{I(c_1)+1}} Q(t)|u(t)|^{\gamma +1}Q_{I(c_1)}^1(t)dt>0. \end{aligned}$$

It is again a contraction with our assumption. The proof when *x*(*t*) is eventually negative is analogous by repeating a similar argument on the interval $$[c_2,d_2].$$$$\square $$

Following Kong (1999) and Philos (1989), we introduce a class of functions: $D= \{(t,s):t_0\le s\le t\},\, H_1, H_2 \in C^{1}(D, {\mathbb {R}}).$ A pair of functions $(H_1, H_2)$ is said to belong to a function class $\mathcal {H},$ if $H_1(t, t) = H_2(t, t) = 0,\, H_1(t, s)> 0, H_2(t, s) > 0$ for $t > s$ and there exist $h_1,h_2\in L_{loc}(D,{\mathbb {R}})$ such that

28 $$\begin{aligned} \frac{\partial H_1(t, s)}{\partial t}= h_1(t, s)H_1(t, s), \quad \,\, \frac{\partial H_2(t, s)}{\partial s}=- h_2(t, s)H_2(t, s). \end{aligned}$$

For $$\lambda \in (c_j,d_j),\,j= 1, 2,$$

$$\begin{aligned} \Gamma _{1,j}= & {} \int _{c_j}^{\tau _{I(c_j)+1}}H_{1}(t,c_j) Q(t)Q_{I(c_j)}^1(t)dt+\sum \limits _{k=I(c_j)+1}^{I(d_j)-1}\int _{\tau _k}^{\tau _{k+1}}H_{1}(t,c_j) Q(t)Q_{k}^{1}(t)dt\\ &+\int _{\tau _{I(d_j)}}^{d_j}H_{1}(t,c_j)Q(t)Q_{_{I(d_j)}}^{1}(t)dt \\ &-\frac{1}{(\gamma +1)^{\gamma +1}}\int _{c_j}^{\lambda _j}r(t)H_{1}(t,c_j)\left| h_1(t,c_j)-\frac{p(t)}{r(t)}\right| ^{\gamma +1}dt, \end{aligned}$$

and

$$\begin{aligned} \Gamma _{2,j}= & {} \int _{\lambda _j}^{\tau _{I(\lambda _j)+1}}H_{2}(d_j,t) Q(t)Q_{I(\lambda _j)}^1(t)dt+\sum \limits _{k=I(\lambda _j)+1}^{I(d_j)-1}\int _{\tau _k}^{\tau _{k+1}}H_{2}(d_j,t) Q(t)Q_{k}^{1}(t)dt \\ &+\int _{\tau _{I(d_j)}}^{d_j}H_{2}(d_j,t)Q(t)Q_{_{I(d_j)}}^{1}(t)dt \\ &-\frac{1}{(\gamma +1)^{\gamma +1}}\int ^{d_j}_{\lambda _j}r(t)H_{2}(t,c_j)\left| h_1(d_j,t)-\frac{p(t)}{r(t)}\right| ^{\gamma +1}dt. \end{aligned}$$

### **Theorem 2**

*Suppose that for any*$T>0,$*there exist*$c_j,d_j,\,j=1,2 ,\lambda \notin \{\tau _k\}$*such that*$c_1<\lambda _1<d_1\le c_2<\lambda _2<d_2$*and* (8) *holds*. *If there exists*$(H_1, H_2)\in \mathcal {H}$*such that*

29 $$\begin{aligned} \frac{1}{H_1(\lambda _1,c_1)}\Gamma _{1,1}+\frac{1}{H_2(d_1,\lambda _1)} \Gamma _{2,1}>\Lambda (H_1,H_2;c_j ,d_j) \end{aligned}$$

*where*

30 $$\begin{aligned} \Lambda (H_1,H_2;c_j ,d_j) =-\left[ \frac{r_j}{H_1(\lambda _ j, c_j)}\Omega _{c_j}^{\lambda _j} [H_1(.,c_j)]+ \frac{r_j}{H_2(d_j,\lambda _ j)}\Omega _{\lambda _j}^{d_j} [H_2(d_j,.)]\right] , \end{aligned}$$

*then every solution of Eq.* (1) *is oscillatory*.

### *Proof*

Let *x*(*t*) be a non-oscillatory solution of Eq. (1). Proceeding as in proof of Theorem 1, we get (13) and (14). Noticing whether or not there are impulsive moments in $[c_1, \lambda _1]$ and $[\lambda _1, d_1],$ we should consider the following four cases, namely: $I(c_1)<I(\lambda _1)<I(d_1);I(c_1)=I(\lambda _1)<I(d_1);I(c_1)<I(\lambda _1)=I(d_1)$ and $I(c_1)=I(\lambda _1)=I(d_1).$ Moreover, in the discussion of the impulse moments of $x(t-\delta ),$ it is necessary to consider the following two cases: $\tau _{_{I(\lambda _j)}+\delta }>\lambda _j$ and $\tau _{_{I(\lambda _j)}+\delta }\le \lambda _j.$ Here we only consider the case $I(c_1)<I(\lambda _1)<I(d_1);$ with $\tau _{_{I(\lambda _j)}+\delta }>\lambda _j.$ For the other cases, similar conclusions can be obtained.

For this case there are impulsive moments $\tau _{_I(c_1)}+1, \tau _{_I(c_1)}+2,\ldots ,\tau _{_{I(\lambda _1)}}$ in $[c_1, d_1]$ and $\tau _{_{I(\lambda _1)+1}},\tau _{_{I(\lambda _1)+2}},\ldots , \tau _{_{I(d_1)}}$ in $[\lambda _1, d_1].$ Multiplying both sides of (13) by $H_{1}(t,c_1)$ and integrating it from $c_1$ to $\lambda _1,$ we have

$$\begin{aligned} \int _{c_1}^{\lambda _1}H_{1}(t,c_1)Q(t)\frac{x^{\gamma }(t-\delta )}{x^{\gamma }(t)}dt&\le -\int _{c_1}^{\lambda _1}H_{1}(t,c_1)\omega '(t)dt\\ {}& \quad- \int _{c_1}^{\lambda _1}\frac{p(t)\omega (t)}{r(t)}H_{1}(t,c_1)dt- \int _{c_1}^{\lambda _1}\frac{\gamma |\omega (t)|^{\frac{\gamma +1}{\gamma }}}{(r(t))^{\frac{1}{\gamma }}}H_{1}(t,c_1)dt. \end{aligned}$$

Applying integration by parts on first integral of R.H.S of last inequality, we get

$$\begin{aligned}&\int _{c_1}^{\lambda _1}H_{1}(t,c_1)Q(t)\frac{x^{\gamma }(t-\delta )}{x^{\gamma }(t)}dt\\ &\quad \le -\sum \limits _{k=I(c_1)+1}^{I(d_1)}H_{1}(\tau _k,c_1)\left( \frac{a_k^\gamma -b_k^\gamma }{a_k^\gamma }\right) \omega (\tau _k)-H_{1}(\lambda _1,c_1)\omega (\lambda _1)\\ &\quad\quad+\left(\int _{c_1}^{\tau _{_{I(c_1)+1}}}+\sum \limits _{k=I(c_1)+1}^{I(d_1)-1} \int _{\tau _k}^{\tau _{k+1}} +\int _{\tau _{_{I(d_1)}}}^{\lambda _1}\right)\left[ h_1(t,c_1)\omega (t)\right. \\  &\left. \quad\quad -\frac{p(t)}{r(t)}\omega (t)- \frac{\gamma |\omega (t)|^{\frac{\gamma +1}{\gamma }}}{(r(t))^{\frac{1}{\gamma }}}\right] H_{1}(t,c_1)dt\\ &\quad \le -\sum \limits _{k=I(c_1)+1}^{I(d_1)}H_{1}(\tau _k,c_1)\left( \frac{a_k^\gamma -b_k^\gamma }{a_k^\gamma }\right) \omega (\tau _k)-H_{1}(\lambda _1,c_1)\omega (\lambda _1)\\ & \quad \quad +\left(\int _{c_1}^{\tau _{_{I(c_1)+1}}}+\sum \limits _{k=I(c_1)+1}^{I(d_1)-1} \int _{\tau _k}^{\tau _{k+1}} +\int _{\tau _{_{I(d_1)}}}^{\lambda _1}\right)\left[ \Big |h_1(t,c_1)\omega (t)\right. \\ &-\left. \left. \frac{p(t)}{r(t)}\right| |\omega (t)|- \frac{\gamma |\omega (t)|^{\frac{\gamma +1}{\gamma }}}{(r(t))^{\frac{1}{\gamma }}}\right] H_{1}(t,c_1)dt. \end{aligned}$$

Using (7) with $A=\left| h_1(t,c_1)-\frac{p(t)}{r(t)}\right| ,\,B=\frac{\gamma }{r(t)^{\frac{1}{\gamma }}}\,\ ,y=|\omega (t)|$ in the last inequality, we have

31 $$\begin{aligned} &\int _{c_1}^{\lambda _1}H_{1}(t,c_1)Q(t)\frac{x^{\gamma }(t-\delta )}{x^{\gamma }(t)}dt\nonumber \\ & \quad \le -\sum \limits _{k=I(c_1)+1}^{I(d_1)}H_{1}(\tau _k,c_1)\left( \frac{a_k^\gamma -b_k^\gamma }{a_k^\gamma }\right) \omega (\tau _k)- H_{1}(\lambda _1,c_1)\omega (\lambda _1)\nonumber \\ &\quad\quad +\frac{1}{(\gamma +1)^{\gamma +1}}\int _{c_1}^{\lambda _1}r(t)H_{1}(t,c_1)\left| h_1(t,c_1)-\frac{p(t)}{r(t)}\right| ^{\gamma +1}dt. \end{aligned}$$

Similar to the proof of Theorem 1, we need to divide the integration interval $[c_1,\lambda _1]$ into several subintervals for estimating the function $\frac{x(t-\delta )}{x(t)}.$ Now,

32 $$\begin{aligned}&\int _{c_1}^{\lambda _1}H_{1}(t,c_1)Q(t)\frac{x^{\gamma }(t-\delta )}{x^{\gamma }(t)}dt\ge \int _{c_1}^{\tau _{I(c_1)+1}}H_{1}(t,c_1) Q(t)Q_{I(c_1)}^1(t)dt\nonumber \\ &\quad +\sum \limits _{k=I(c_1)+1}^{I(d_1)-1}\int _{\tau _k}^{\tau _{k+1}}H_{1}(t,c_1) Q(t)Q_{k}^{1}(t)dt+\int _{\tau _{I(d_1)}}^{d_1}H_{1}(t,c_1)Q(t)Q_{_{I(d_1)}}^{1}(t)dt. \end{aligned}$$

From (31) and (32),we obtain

33 $$\begin{aligned}&\int _{c_1}^{\tau _{I(c_1)+1}}H_{1}(t,c_1) Q(t)Q_{I(c_1)}^1(t)dt+\sum \limits _{k=I(c_1)+1}^{I(d_1)-1}\int _{\tau _k}^{\tau _{k+1}}H_{1}(t,c_1) Q(t)Q_{k}^{1}(t)dt\nonumber \\ &\quad\quad +\int _{\tau _{I(d_1)}}^{d_1}H_{1}(t,c_1)Q(t)Q_{_{I(d_1)}}^{1}(t)dt -\frac{1}{(\gamma +1)^{\gamma +1}}\int _{c_1}^{\lambda _1}r(t)H_{1}(t,c_1)\left| h_1(t,c_1)-\frac{p(t)}{r(t)}\right| ^{\gamma +1}dt\nonumber \\ &\quad \le \sum \limits _{k=I(c_1)+1}^{I_{_{(\lambda _1)}}}H_{1}(\tau _k,c_1) \left( \frac{a_k^\gamma -b_k^\gamma }{a_k^\gamma }\right) \omega (\tau _k)-H_{1}(\lambda _1,c_1)\omega (\lambda _1). \end{aligned}$$

On the other hand multiplying both sides of (13) by $H_2(d_1,t)$ and then integrating from $\lambda _1$ to $d_1$ and using similar analysis to the above, we can obtain

34 $$\begin{aligned}&\int _{\lambda _1}^{\tau _{I(\lambda _1)+1}}H_{2}(d_1,t) Q(t)Q_{I(\lambda _1)}^1(t)dt+\sum \limits _{k=I(\lambda _1)+1}^{I(d_1)-1}\int _{\tau _k}^{\tau _{k+1}}H_{2}(d_1,t) Q(t)Q_{k}^{1}(t)dt\nonumber \\ &\quad \quad  +\int _{\tau _{I(d_1)}}^{d_1}H_{2}(d_1,t)Q(t)Q_{_{I(d_1)}}^{1}(t)dt -\frac{1}{(\gamma +1)^{\gamma +1}}\int ^{d_1}_{\lambda _1}r(t)H_{2}(t,c_1)\left| h_1(d_1,t)-\frac{p(t)}{r(t)}\right| ^{\gamma +1}dt\nonumber \\ &\quad \le -\sum \limits _{k=I(\lambda _1)+1}^{I(d_1)}H_{2}(d_1,\tau _k) \left( \frac{a_k^\gamma -b_k^\gamma }{a_k^\gamma }\right) \omega (\tau _k)-H_{2}(d_1,\lambda _1)\omega (\lambda _1). \end{aligned}$$

Dividing (33) and (34) by $H_1(\lambda _1,c_1)$ and $H_2(d_1,\lambda _1)$ respectively and adding them, we get

35 $$\begin{aligned}&\frac{1}{H_1(\lambda _1,c_1)}\Gamma _{1,1}+\frac{1}{H_2(d_1,\lambda _1)} \Gamma _{2,1}\nonumber \\ &\le -\Bigg (\frac{1}{H_1(\lambda _1,c_1)}\sum \limits _{k=I(c_1)+1}^{I(d_1)}H_{1}(\tau _k,c_1) \left( \frac{a_k^\gamma -b_k^\gamma }{a_k^\gamma }\right) \omega (\tau _k)\nonumber \\ & \quad +\frac{1}{H_2(d_1,\lambda _1)}\sum \limits _{k=I(\lambda _1)+1}^{I(d_1)}H_{2}(d_1,\tau _k) \left( \frac{a_k^\gamma -b_k^\gamma }{a_k^\gamma }\right) \omega (\tau _k)\Bigg ).\\ \nonumber \end{aligned}$$

Using the same method as in (27), we obtain

36 $$\begin{aligned} -\sum \limits _{k=I(c_1)+1}^{I(d_1)}H_{1}(\tau _k,c_1) \left( \frac{a_k^\gamma -b_k^\gamma }{a_k^\gamma }\right) \omega (\tau _k)\le & {} -r_1\Omega _{c_1}^{\lambda _1}[H_1(.,c_1)]\nonumber \\ -\sum \limits _{k=I(\lambda _1)+1}^{I(d_1)}H_{2}(d_1,\tau _k) \left( \frac{a_k^\gamma -b_k^\gamma }{a_k^\gamma }\right) \omega (\tau _k)\le & {} -r_2\Omega ^{d_1}_{\lambda _1}[H_2(d_1,.)].\\ \nonumber \end{aligned}$$

From (33) and (36), we obtain

$$\begin{aligned} \frac{1}{H_1(\lambda _1,c_1)}\Gamma _{1,1}+\frac{1}{H_2(d_1,\lambda _1)} \Gamma _{2,1}&\le -\left( r_1\Omega _{c_1}^{\lambda _1}[H_1(.,c_1)] +r_2\Omega ^{d_1}_{\lambda _1}[H_2(d_1,.)]\right) \\ &\le \Lambda (H_1,H_2;c_j ,d_j) \end{aligned}$$

which is a contradiction to the condition (29). When $x(t) < 0,$ we choose interval $[c_2, d_2]$ to study Eq. (1). The proof is similar and hence omitted. Now the proof is complete. $\square$

### *Remark 2*

When $p(t)= 0,$ Eq. (1) reduces to the equation studied by Guo et. al (2012b). Therefore our Theorem 1 provides an extension of Theorem 2.3 with $\rho (t)=1$ to damped impulsive differential equation.

### *Remark 3*

When $\delta = 0,$ that is, the delay disappears and our results reduces to that of Theorem 2.1 and Theorem 1 with $\rho (t)=1$ in Pandian and Purushothaman (2012).

### *Remark 4*

When $p(t)=0$ and $\gamma =1$ our Theorem 1 is a generalization of Theorem 2.2 in Li and Cheung (2013).

### *Remark 5*

When the impulse is disappear, i.e., $a_k= b_k=1$ for all $k= 1,2,\ldots ,$ the delay term $\delta =0$ and $p(t)=0$ Eq. (1) reduces to the situation studied in Hassan et al. (2011). Therefore our Theorem 1 extends Theorem 2.1 of Hassan et al. (2011).

### *Example 1*

Consider the following impulsive differential equation

37 $$\begin{aligned} \left\{ \begin{array}{ll} (((2+cos t)x'(t)^{\frac{9}{5}}))'+(1+\sin t) (x'(t))^{\frac{9}{5}}+ m_1 (\cos t)|x(t-\frac{\pi }{8})|^{\frac{3}{2}}x(t-\frac{\pi }{8})\\ \quad\quad +m_2(\cos t)|x(t-\frac{\pi }{8})|^{\frac{1}{2}}x(t-\frac{\pi }{8})=\sin 2t,\,t\ne 2k\pi \pm \frac{\pi }{4};\\ x(\tau _k^+)= \frac{1}{3}x(\tau _{k}), x^{'}(\tau _k^+)=\frac{2}{3}x'(\tau _{k}),\,\tau _k=2k\pi \pm \frac{\pi }{4},\, k=1,2, \ldots \\ \end{array} \right. \end{aligned}$$

Here $r(t)=2+cos t,\, p(t)=1+\sin t,\, q_1(t)=m_1\cos t,\, q_2(t)=m_2 \ cos t,\, e(t)=\sin 2t,\, \gamma =\frac{9}{5},\, \alpha _1=\frac{5}{2},\,\alpha _2=\frac{3}{2}$ and $m_1,\,m_2$ are positive constants. Also $\delta =\frac{\pi }{8},\, \tau _{k+1}-\tau _k= \pi /2>\pi /8.$ For any $T>0,$ we can choose *k* large enough such that $T<c_1=4k\pi -\frac{\pi }{2}<d_1=4k\pi$ and $c_2=4k\pi +\frac{\pi }{8}<d_2=4k\pi +\frac{\pi }{2},\, k=1,2 \ldots .$ Then there is an impulsive moment $\tau _k=4k\pi -\frac{\pi }{4}$ in $[c_1, d_1]$ and an impulsive moment $\tau _{k+1}= 4k\pi +\frac{\pi }{4}$ in $[c_2, d_2].$ Now choose $\eta _0=1/5,\, \eta _1=3/5,\, \eta _2=1/5,$ therefore

$$\begin{aligned} Q(t)= 5 \frac{2^{\frac{1}{5}}}{3^{\frac{3}{5}}}(m_1)^{\frac{3}{5}}(m_2)^{\frac{1}{5}}|\cos t||\sin t|^{\frac{1}{5}} \end{aligned}$$

If we take $u1(t)=u_2(t)=\sin 4t,\,\tau _{_{I(c_1)}}=4k\pi -\frac{7}{4}\pi ,\, \tau _{_{I(d_1)}}=4k\pi -\frac{\pi }{4},$ then by a simple calculation, the left side of Eq. (9) is the following:

$$\begin{aligned}& \int _{c_1}^{d_1}\frac{r(t)}{(\gamma +1)^{\gamma +1}}\Big ((\gamma +1)|u'(t)|-\frac{p(t)|u(t)|}{r(t)}\Big )^{\gamma +1}dt- \int _{c_1}^{\tau _{I(c_1)+1}} Q(t)|u(t)|^{\gamma +1}Q_{I(c_1)}^1(t)dt\\ &\quad\quad -\sum \limits _{k=I(c_1)+1}^{I(d_1-1)} \int _{\tau _k}^{\tau _{k+1}} Q(t)|u(t)|^{\gamma +1}Q_{k}^{1}(t)dt -\int _{\tau _{I(d_1)}}^{d_1}Q(t)|u(t)|^{\gamma +1}Q_{_{I(d_1)}}^{1}(t)dt\\ &\quad \ge \frac{1}{(\frac{14}{5})^{\frac{14}{5}}}\int _{4k\pi -\frac{\pi }{2}}^{4k\pi }(2+\cos t)\Big (\frac{56}{5}|\cos 4t|-\frac{(1+\sin t)|\sin 4t|}{(2+\cos t)}\Big )^{\frac{14}{5}}dt\\ &\quad\quad - \int _{4k\pi -\frac{\pi }{2}}^{4k\pi -\frac{\pi }{4}} Q(t) |\sin 4t|^{\frac{14}{5}} \left( \frac{t-\frac{\pi }{8}-4k\pi +\frac{7\pi }{4}}{t-4k\pi +\frac{7\pi }{4}}\right) ^{\frac{9}{5}}dt\\ &\quad \quad  -\int _{4k\pi -\frac{\pi }{4}}^{4k\pi -\frac{\pi }{8}} Q(t)|\sin 4t|^{\frac{14}{5}}\left( \frac{t-4k\pi +\frac{\pi }{4}}{a_{_{I(c_{1})+1}}(t+\frac{\pi }{8}-4k\pi +\frac{\pi }{4})}\right) ^{\frac{9}{5}}dt\\ &\quad \quad -\int _{4k\pi -\frac{\pi }{8}}^{4k\pi }Q(t) |\sin 4t|^{\frac{14}{5}}\left( \frac{t-\frac{\pi }{8}-4k\pi +\frac{\pi }{4}}{t-4k\pi +\frac{\pi }{4}}\right) ^{\frac{9}{5}}dt\\ &\approx (m_1)^{\frac{3}{5}}(m_2)^{\frac{1}{5}} (1.5196)-0.6739. \end{aligned}$$

Since $I(c_1)=k-1,\, I(d_1)=k,r_1=3,\,$ we have

$$\begin{aligned} r_1\Omega _{c_1}^{d_1}[|u(t)|^{\gamma +1}] &=  3|\sin 4(\tau _k)|^{\frac{14}{5}}\left( \frac{a_k^{\frac{9}{5}}-b_k^{\frac{9}{5}}}{a_k^{\frac{9}{5}}}\right) \\ &= 0. \end{aligned}$$

The condition (9) is satisfied in $[c_1, d_1]$ if

38 $$ (m_1)^{\frac{3}{5}}(m_2)^{\frac{1}{5}}(1.5196)< 0.6739 $$

Similarly, we can show that for $t\in [c_2, d_2],$ the condition (9) is satisfied if

39 $$(m_1)^{\frac{3}{5}}(m_2)^{\frac{1}{5}}(0.7553)< 0.5233 $$

Since the condition (38) is weaker than (39) we can choose the constants $m_1,\, m_2$ small enough such that (39) holds. Hence by Theorem 1 every solution of Eq. (37) is oscillatory. In fact for $m_1=1/5,\, m_2=1/6,$ every solution of Eq. (37) is oscillatory.

### *Remark 6*

The result obtained in Guo et al. (2012a, b, 2014) and Erbe et al. (2010) cannot be applied to Example 1, since the results in Guo et al. (2012a) can be applicable only to equations having only one nonlinear term and the results in Guo et al. (2012b), Guo et al. (2014), Erbe et al. (2010) can be applied to equations without damping term. Therefore our results extent and compliment to Guo et al. (2012a, b, 2014), Hassan et al. (2011), Li and Cheung (2013), Pandian and Purushothaman (2012) and Erbe et al. (2010).

## Conclusion

In this paper we have obtained interval oscillation criteria for Eq. (1) which extend and generalize some known results in Guo et al. (2012a), Li and Cheung (2013), Hassan et al. (2011) and Özbekler and Zafer (2011), Pandian and Purushothaman (2012).
